# L-DOPA Promotes Post-Treatment Neurovascular and Synaptic Homeostasis in Early Diabetic Retinopathy

**DOI:** 10.1167/iovs.67.8.42

**Published:** 2026-07-20

**Authors:** Eli Chlan, Chenxing Li, Katie L. Bales, Levi B. Wood, Machelle T. Pardue

**Affiliations:** 1 Department of Ophthalmology and Neuroscience Program, Emory University, Atlanta, Georgia, United States; 2 Joseph Maxwell Cleland Atlanta VA Medical Center, Atlanta, Georgia, United States; 3 Wallace H. Coulter Department of Biomedical Engineering, Emory University Georgia Institute of Technology, Atlanta, Georgia, United States; 4 George W. Woodruff School of School of Mechanical Engineering and Parker H. Petit Institute for Bioengineering and Bioscience, Georgia Institute of Technology, Atlanta, Georgia, United States

**Keywords:** diabetic retinopathy, dopamine, neuroprotection, transcriptomics

## Abstract

**Purpose:**

Although previous work has shown a post-treatment protective effect of levodopa (L-DOPA) on retinal function in early-stage diabetic retinopathy (DR) in humans, its underlying biology is unknown. This study investigated L-DOPA's post-treatment functional protection with transcriptional changes in the diabetic murine retina.

**Methods:**

Assessing retinal and visual function with electroretinography (ERG) and optomotor response (OMR), functional deficits were confirmed in streptozotocin (STZ)-induced diabetic mice. Control and diabetic mice were then treated with continuous L-DOPA/carbidopa (four weeks), L-DOPA/carbidopa (two weeks) followed by washout (two weeks), or vehicle (four weeks). Functional assessments were repeated during the final two weeks, alongside assessment of flicker-evoked retinal vasodilation. After bulk RNA sequencing of retinal tissue, differential gene expression analysis alongside weighted gene co-expression network analysis were performed to determine disease- and treatment-sensitive changes in retinal gene co-expression that correlated with functional protection.

**Results:**

After L-DOPA treatment in diabetic mice, ERG oscillatory potential timing and OMR performance were protected for at least two weeks past treatment end. Flicker-induced venule vasodilation also maintained post-treatment improvement, with protective trends in arteriole vasodilation. Differentially expressed genes were comparable between diabetic mice experiencing L-DOPA washout versus continued L-DOPA treatment. Gene co-expression network analysis identified distinct modules across L-DOPA–treated diabetic mice associated with synaptic function and cytoskeletal organization that correlated with functional protection.

**Conclusions:**

These findings demonstrate that L-DOPA restores retinal neurovascular function with post-treatment effects in early DR and links this protection to transcriptional programs supporting synapse activity and structural integrity.

Diabetic retinopathy (DR) has historically been defined as a retinal microvascular complication resulting from diabetes mellitus (DM) and has more recently been identified as a neurovascular disease.^1^^–^^3^ One-third of diabetic patients develop DR, with a further third of DR patients experiencing vision-threatening DR.^4^ Clinical detection and classification of DR relies on visualizing the development of vascular manifestations, such as microaneurysms, venous beading, retinal and vitreous hemorrhages, gliosis, or retinal neovascularization.^2^^,^^5^^,^^6^ Although convenient for diagnosis, ongoing research has revealed neuronal and vascular dysfunction that precede these large-scale, vision-threatening vascular changes.^7^^–^^12^

These early deficits in the diabetic retina span the neurovascular unit (NVU)—an intricate coupling of neurons, glia, immune cells, and vasculature within the central nervous system. Unlike the rest of the eye, the retina lacks autonomic innervation and relies on the NVU for autoregulation of retinal circulation.^13^ In preclinical DR, changes across cell types of the NVU include delay in rod-specific neuronal signaling,^7^^–^^11^ reduction of neuronal synaptic connectivity,^12^^,^^14^ gliosis by Müller cells,^12^ reduction of blood flow and basal vessel diameter,^15^^–^^17^ and increasing interactions of glia (e.g., microglia) with retinal vasculature.^18^ Several studies show that individual deficits within the NVU of the diabetic retina disrupt the function of its associated components.^19^^–^^21^ Given this interdependence, identifying treatments for DR that target these early functional deficits in the retina may be critical to deterring progression toward vision-threatening vascular complications.

In the retina, dopamine is a critical neuromodulator that shapes gap junction coupling and intrinsic ionic conductance, facilitating dynamic modulation of retinal circuitry to optimize performance under different lighting conditions.^22^^–^^24^ Retinal dopamine levels are decreased in diabetic rodent models, with reduced dopamine concomitant with early functional changes, such as amacrine cell-derived oscillatory potential implicit timing (OP IT) delays.^8^^,^^25^ Although dopamine receptor expression appears preserved in the diabetic retina, reduced responsiveness to D1R and D4R agonists suggests impaired downstream dopaminergic signaling.^26^^,^^27^ Importantly, restoring dopamine in diabetic rodent models by administration of dopamine's precursor, levodopa (L-DOPA), has been protective against OP IT delays and visual function deficit when given at hyperglycemic onset,^8^^,^^11^ or administered after OP IT delays have been detected.^9^ Strikingly, translation to diabetic patients has shown that L-DOPA is restorative for OP IT delays and that this neuroprotective effect is maintained for at least two weeks beyond the treatment window.^10^

However, biological underpinnings relevant to L-DOPA's post-treatment functional neuroprotection in the diabetic retina remain unexplored. Here, we show L-DOPA benefits retinal neurovascular coupling and replicate L-DOPA's lasting protective effects on retinal function in diabetic mice. Furthermore, we establish L-DOPA-induced transcriptional changes in the diabetic retina, with gene networks spanning synapse signaling and cytoskeletal components correlative with neurovascular functional protection maintained after L-DOPA washout.

## Material and Methods

### Animals

Three-month-old male C57BL/6J mice (Jackson Laboratories, Bar Harbor, ME, USA) were housed in the animal facility at the Joseph Maxwell Cleland Atlanta Veterans Affairs Medical Center (Decatur, GA) under a 12:12-hour (light:dark) cycle with food and water ad libitum. Hyperglycemia was induced via daily intraperitoneal injection of low-dose STZ (50 mg/kg, in citrate buffer, pH 4.5) for five consecutive days; controls received equal volumes of citrate buffer. Blood glucose was monitored via tail vein sampling using a handheld glucose meter (Freestyle; Abbott, Abbott Park, IL, USA), with hyperglycemia confirmed by two consecutive readings >250 mg/dL. Blood glucose and body weight were monitored weekly throughout the study. In cases of successive weight reduction (>10% body weight) following hyperglycemia, a single low-dose insulin injection (1:10 insulin in lactated Ringer's, intraperitoneally) was administered to stabilize weight loss without hypoglycemia. All procedures were approved by the Institutional Animal Care and Use Committee of the Atlanta Veterans Affairs Medical Center and performed in full accordance with the ARVO Statement for the Use of Animals in Ophthalmic and Vision Research.

### Experimental Design

**Figure 1.**
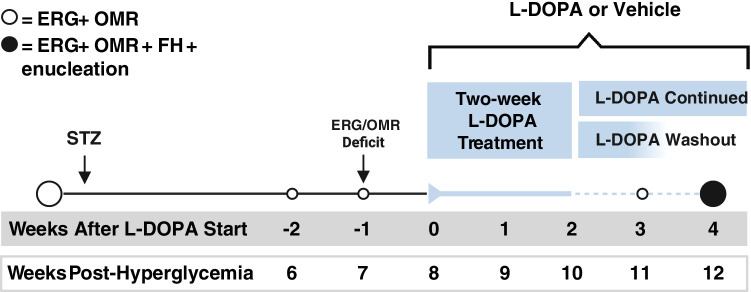
Timeline of experimental design for L-DOPA treatment in the diabetic mouse. ERGs and OMRs were taken at baseline before mice were made diabetic via STZ or kept as controls. After ERG/OMR deficits were detected, mice received two-week L-DOPA or vehicle treatment, followed by two weeks of either continued L-DOPA/vehicle treatment or no treatment (“washout”). At the final timepoint, ERGs, OMRs, and functional hyperemia (FH) were measured and followed by enucleation.

Baseline retinal and visual function were assessed by electroretinography (ERG) and optomotor response (OMR), respectively (Fig. 1), before diabetic induction. Starting four weeks after hyperglycemia, ERGs and OMRs were collected weekly to identify functional deficits. After deficit detection, mice were randomized to daily oral L-DOPA or vehicle (Veh) for two weeks. L-DOPA–treated mice were subsequently assigned to continued L-DOPA treatment (Cont) or vehicle (Wash) to induce a washout phase, with washout timing designed to mirror previous clinical findings.^10^ Group allocation was: Ctrl+Veh (*n* = 9), Ctrl+Cont (*n* = 9), Ctrl+Wash (*n* = 6), DM+Veh (*n* = 12–15), DM+Cont (*n* = 12–15), and DM+Wash (*n* = 7–11). During washout, ERGs and OMRs were collected weekly. In the final week, functional hyperemia measurements before euthanasia (cervical dislocation), and retinas from left eyes were collected and flash-frozen for RNA isolation.

### Oral L-DOPA Treatment

L-DOPA (Sigma-Aldrich, St. Louis, MO, USA) was suspended in sweetened condensed milk (California Farms, Santini Foods, Inc., San Lorenzo, CA, USA) and fed to mice under dim-light conditions in individual static cages. On testing days, L-DOPA treatment was fed after functional assessments. As previously described,^28^ L-DOPA treatment was prepared fresh daily under dim-light conditions with L-DOPA (20 mg/kg at 20 mg/mL) and carbidopa monohydrate (5 mg/kg at 5 mg/mL), the latter preventing premature peripheral L-DOPA oxidation.^29^^,^^30^ One cohort was treated with L-DOPA/carbidopa at one-quarter of the original dosage throughout the treatment window, showed no functional differences in continuously treated groups, and was combined with the original dosage for downstream analysis.

### ERGs

For retinal function assessment, dark-adapted ERG recordings were acquired as previously described.^8^^,^^9^^,^^11^ Using a five-step flash protocol, white light stimuli within a dark-adapted series were presented (−3.0, −1.7, −0.6, 1.5, 1.9 log cd s/m^2^). Oscillatory potentials were filtered digitally with a high-pass filter (75–350 Hz) using custom MATLAB software.^31^ Data from only one eye with the largest oscillatory potential amplitude at −3.0 log cd s/m^2^ flash was analyzed at each flash intensity per animal.

### Optomotor Response

Visual function of mice was assessed with a virtual optomotor system (OptoMotry system; Cerebral-Mechanics, Lethbridge, AB, Canada). Briefly, mice were placed on the central pedestal of a virtual reality chamber with four surrounding monitors, as previously described.^8^^,^^9^ After one minute of environmental acclimation, monitors displayed vertical sine wave gratings rotating at 12°/s speed. A staircase paradigm separately probed spatial frequency (SF) (100% contrast) and contrast sensitivity (CS) (0.103 cycles/degree [cyc/deg]) thresholds, with reflexive head movements (tracking) recorded.

### Functional Hyperemia

To assess retinal vascular function, confocal scanning laser ophthalmoscopy (Heidelberg Spectralis; HRA+OCT; Heidelberg Engineer, Carlsbad, CA, USA) was performed during photopic stimulation. After anesthesia and eye preparation as described for ERGs, retinal vessels were visualized via intraperitoneal injection of indocyanine green (ICG; 18.75 mg/kg; IC-Green; Akorn, Lake Forest, IL, USA). A 12 Hz square wave green light stimulus (480-600 nm) was presented through a fiber optic bundle and reflected off a 45° angle prism mirror (TS Cold Mirror; Edmund Optics, Barrington, NJ, USA) to avoid interrupting image acquisition. ICG angiography video was acquired at high resolution with a widefield 55° scan angle. Each recording consisted of a 10 second baseline, 15 seconds flicker stimulation, and 10 seconds post-flicker; trials with motion artifacts were repeated after a two-minute interval.

Flicker-induced vasodilation was quantified from vessel caliber kymographs generated from ICG fundus video sequences (ImageJ; U. S. National Institutes of Health, Bethesda, MD, USA). Two first-order arterioles or venules per eye were randomly selected, and kymographs were derived from cross-sections positioned one optic disc diameter away from the optic nerve. Vessel identity relied on morphology, depth, and alternating pattern.^32^^,^^33^ Vessel diameters during baseline (0-10 seconds) and stimulation (12.5–22.5 seconds) were averaged in a custom MATLAB script and percent dilation was calculated following moving window average smoothing (Mathworks, Natick, MA, USA). Representative venule traces were selected by traces providing percent dilation values closest to the mean of each group.

### RNA Sequencing and Data Preparation

For RNASeq bulk analysis, retinas from left eyes were collected in ambient lighting between 10:00 AM and 2:00 PM, flash-frozen on dry ice, and stored at −80°C. Mice given continuous L-DOPA were administered L-DOPA 15 minutes before enucleation. Total RNA was extracted (RNeasy, catalog no. 74106; Qiagen, Hilden, Germany) via manufacturer's protocol and sent to Admera Health (South Plainfield, NJ, USA) for bulk sequencing and alignment. After quality control (RNA Integrity Number>7), mRNA was prepared with NEB Next Ultra (II) Directional Kit with Poly A Selection and sequenced (2 × 150 bp) using the Illumina platform at 20 million reads/direction. Reads were aligned with STAR Aligner (v2.7.1a) and gene counts/sample were determined with HTseq-count (v0.11.2).

### Differential Gene Expression Analysis

Transcripts with <40 counts were filtered, leaving 13,665 transcripts for downstream analysis. Normalization and differential expression analysis were performed using *DESeq2* R package (v1.49.2) with median-of-ratios normalization and a condition-specific design to fit a negative binomial generalized linear model per gene. Batch effects were corrected using the *RemoveBatchEffect* function from *limma* (v3.65.1). Principle component analysis (PCA) of normalized, batch-corrected data was used for outlier identification via Mahalanobis distance, with samples outside the 95% confidence ellipse in PCA space iteratively removed after two rounds (*n* = 7). Differentially expressed genes (DEGs) were determined using a Benjamini-Hochberg adjusted *P*-value (<0.05) and |log_2_ fold change| (>0.32). Volcano plots were generated using *EnhancedVolcano* (v1.27.0).

### Weighted Gene Co-Expression Network Analysis

Weighted gene co-expression network analysis (WGCNA) was performed using the *WGCNA* R package (v1.73). Counts were normalized with *DESeq2* (v1.49.2) using median-of-ratios with condition and batch in the design, variance-stabilized, and batch-corrected using *limma* (v3.65.1). Lowly expressed genes (<40 counts) were filtered prior to network construction. A signed network was constructed using *biweight midcorrelation* with a soft-tresholding power of 8 (scale-free topology fit R2>0.8). Modules of co-expressed genes were identified via hierarchical clustering (min size = 50, max size = 13,665, deep split = 4) and merged at a cut height of 0.25 using a mean-based topological overlap matrix. Module eigengenes (MEs) per sample were correlated with functional data using Pearson correlation, with |*r*|> 0.3 and unadjusted *P* < 0.05 identifying relevant module-trait associations.

### Gene Ontology Analysis

Gene ontology (GO) enrichment analysis for biological processes was performed for thresholded genes within WGCNA modules (>0.6 kME) using *clusterProfiler* (v4.17.0) and *enricher* function. Gene sets were obtained from the MSigDB database (Mus Musculus, GO:BP) with *msigdbr* (v25.1.1). Benjamini-Hochberg false discovery rate (FDR) adjusted *P*-values (<0.05) identified significance, represented in chord diagrams created with *circlize* (v0.4.16).

### Retinal Cell-Type Percent Enrichment Analysis

To evaluate identity of WGCNA modules, we performed enrichment analysis across NVU class and major retinal cell type. Using the Mouse Retina Cell Atlas,^34^ unique gene sets formed across broad NVU classes (e.g., neuronal, glial, microglial, and vascular; Supplementary Data S5) and major cell type classes (e.g., bipolar, amacrine, ganglion, Muller glia, astrocytes, microglia, endothelial cells, pericytes, rods, and cones; Supplementary Data S6). Genes were filtered for significantly differential expression (log₂ fold change > 0.25, adjusted *P* < 0.05, top 1000 genes per major cell type; top 2000 genes per NVU class), and retained if unique within class. Module percent enrichment was determined by dividing the number of cell type- or NVU class-specific genes by total module hubgenes (>0.7 kME).

### Statistical Analysis of Functional Data

Statistical analysis of functional data was performed using statistical software (GraphPad Prism v10.6.1, GraphPad Software, San Diego, CA, USA). Longitudinal ERG and OMR datasets were analyzed using a two-way mixed effects model, while functional hyperemia measures were analyzed with a one-way ANOVA. All functional measure statistics reported are interaction effects followed by Tukey's multiple comparison test. Statistical significance threshold is indicated by *P < 0.05, **P < 0.01, **P < 0.01, ***P < 0.001, ****P < 0.0001. Significance symbols used in longitudinal data reflects group comparisons: (*) DM+Veh vs. Ctrl+Veh, (†) DM+Veh vs. DM+Cont, (‡) DM+Veh vs. DM+Wash, (§) Ctrl+Veh vs. DM+Cont, (#) Ctrl+Veh vs. DM+Wash. Figures show mean ± SEM for each group.

### Data Availability

The dataset generated and analyzed during this study is publicly available at the National Center for Biotechnology Information Gene Expression Omnibus under the following accession number: GSE318897.

## Results

### Post-Treatment Benefit of L-DOPA Treatment for Rod-Driven Inner Retinal Function in Diabetic Mice

**Figure 2.**
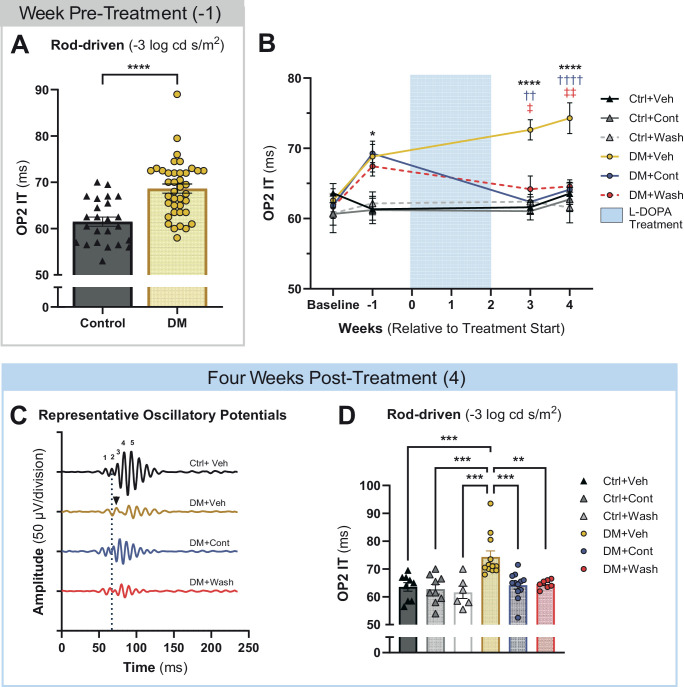
L-DOPA shows post-treatment improvement of rod-driven oscillatory potential (OP) timing. **(A)** Rod-driven OP timing (OP2 IT) was significantly delayed in diabetic mice compared to control mice prior to L-DOPA treatment. **(B)** After two weeks of L-DOPA treatment, DM+Cont and DM+Wash mice consistently showed improved OP2 IT over DM+Veh mice to the final timepoint, whereas DM+Veh OP2 IT grew significantly more delayed. Ctrl+Cont/Wash comparison statistics are not shown here. **(C)** Representative OP waveforms from individual mice at the final experimental timepoint show DM+Veh OP2 IT delay (*black arrowhead*) versus the timing of Ctrl+Veh OP2 IT (*vertical dotted line*). Note OP2 is similar or faster in the Cont and Wash L-DOPA groups. **(D)** Before enucleation, OP2 IT was similar between DM+Cont, DM+Wash and control mice, while significantly delayed in DM+Veh mice compared to all other groups. Ctrl+Veh (*n* = 9), Ctrl+Cont (*n* = 9), Ctrl+Wash (*n* = 6), DM+Veh (*n* = 12–15), DM+Cont (*n* = 12–15), and DM+Wash (*n* =7–11). In 2A, Control represents all mice randomized as Ctrl+Veh, Ctrl, Ctrl+Cont, and Ctrl+wash (*n* = 24). Data shown as mean ± SEM; (*) DM+Veh vs. Ctrl+Veh, (†) DM+Veh vs. DM+Cont, (‡) DM+Veh vs. DM+Wash; **P < 0.01, ***P < 0.001, ****P < 0.0001. Includes assets from BioRender.

Prioritizing clinical relevance, L-DOPA or vehicle treatments were administered to all groups only after retinal and visual functional deficits were detected in hyperglycemic mice (Fig. 1). Hyperglycemia (>250 mg/dL) developed in STZ-administered mice after one week and was confirmed across longitudinal measures (Supplementary Fig. S1). After 7.33 ± 0.67 (mean ± SEM) weeks of hyperglycemia, diabetic mice developed significantly delayed oscillatory potential (OP) implicit timing (IT; Fig. 2A; Ctrl: 61.5 ± 0.98 ms vs. DM: 68.8 ± 1.03 ms, *P* < 0.0001).

After four weeks of continuous L-DOPA treatment following detection of OP delays, DM+Cont mice had significantly improved OP IT compared to DM+Veh (*P* < 0.0001; Figs. 2B–D; Table) and were statistically indistinguishable from Ctrl+Veh OP2 IT (*P* = 0.999; Figs. 2B–D). DM mice treated with L-DOPA for two weeks followed by two weeks of washout (DM+Wash) also reflected control-like timing and maintained improved OP2 IT over DM+Veh (*P* = 0.004). Similar to our previous findings,^8^^,^^11^ improvement in OP timing in DM+Cont and DM+Wash mice was exclusively rod-driven, with no significant differences in OP IT elicited by brighter flash stimuli (Supplementary Fig. S2A). Furthermore, non-DM Ctrl mice receiving L-DOPA under continued (Ctrl+Cont) or washout (Ctrl+Wash) conditions showed no change to amplitude or timing across OPs, B-wave, and A-wave (Figs. 2B–D; Supplementary Fig. S2).

**Table.** In vivo Functional Assessment From the Final Timepoint (Four Weeks After Treatment) Assessed Across DM Condition And L-DOPA Treatment Groups: Ctrl+Veh (*n* = 8), Ctrl+Cont (*n* = 9), Ctrl+Wash (*n* = 6), DM+Veh (*n* = 12), DM+Cont (*n* = 12), and DM+Wash (*n* = 7)

	Control			Diabetic				
	Ctrl+Veh	Ctrl+Cont	Ctrl+Wash	DM+Veh	DM+Cont	DM+Wash	*F*(ANOVA)	MC
OP2 IT (ms)	63.56 ± 1.53	62.72 ± 1.73	61.58 ± 2.18	74.29 ± 2.19	64.13 ± 1.38	64.57 ± 0.59	*F*(15, 156) = 3.498****	****,††††,‡‡
SF (cyc/deg)	0.402 ± 0.001	0.403 ± 0.001	0.401 ± 0.002	0.341 ± 0.004	0.385 ± 0.002	0.374 ± 0.003	*F*(15, 156) = 39.07****	****,††††,‡‡‡‡
Contrast sensitivity (a.u.)	8.754 ± 0.153	8.846 ± 0.112	8.791 ± 0.300	4.778 ± 0.210	7.259 ± 0.170	6.551 ± 0.266	*F*(15, 156) = 38.54****	****,††††,‡‡‡‡
Arteriole vasodilation	3.69% ± 0.90%	4.05% ± 0.50%	4.09% ± 0.50%	1.17% ± 0.26%	3.44% ± 0.47%	3.32% ± 0.97%	*F*(5, 48) = 4.088****	****,††††,‡‡‡
Venule vasodilation	3.82% ± 0.38%	3.67% ± 0.22%	3.35% ± 0.26%	1.22% ± 0.24%	3.03% ± 0.24%	3.07% ± 0.27%	*F*(5, 48) = 14.49**	*,†

Mean ± SEM for final functional timepoint. *F*(ANOVA) indicates interaction effect for mixed-effects model (OP2 IT, SF, OMR) and main effect for one-way ANOVAs (vasodilation). DM+Veh multiple comparisons (MC) indicated vs. Ctrl+Veh (*), versus DM+Cont (†), vs. DM+Wash (‡).

*P < 0.05; **P < 0.01; **P < 0.01; ***P < 0.001; ****P < 0.0001.

### L-DOPA Induced Lasting Visual Function Protection in the Diabetic Retina

**Figure 3.**
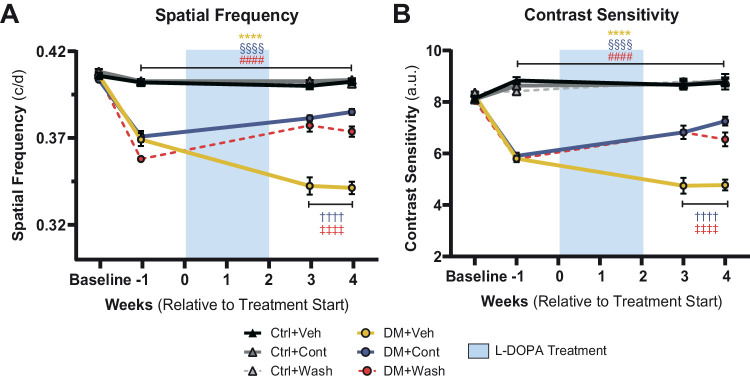
Visual function improvement following L-DOPA treatment in diabetic mice. **(A)** Diabetic mice showed significant reductions in SF thresholds before L-DOPA treatment (−1 week treatment). After two weeks of L-DOPA treatment, DM+Cont and DM+Wash mice demonstrated significantly improved SF thresholds compared to DM+Veh, with dysfunction plateauing compared to continued SF decline in DM+Veh. **(B)** As seen in SF, contrast sensitivity (CS) thresholds significantly declined in diabetic mice before treatment started. L-DOPA treatment induced significantly improved CS thresholds and a similar plateauing effect in DM+Cont and DM+Wash mice compared to DM+Veh mice. Ctrl+Veh (*n* = 9), Ctrl+Cont (*n* = 9), Ctrl+Wash (*n* = 6), DM+Veh (*n* = 12–15), DM+Cont (*n* = 12–15), and DM+Wash (*n* = 7–11). Data shown as mean ± SEM; (*) DM+Veh vs. Ctrl+Veh, (†) DM+Veh vs. DM+Cont, (‡) DM+Veh vs. DM+Wash, (§) Ctrl+Veh vs. DM+Cont, (#) Ctrl+Veh vs. DM+Wash; ****P < 0.0001. Includes assets from BioRender.

Similar to the retinal function deficit detected by ERGs, DM mice had reduced SF (Ctrl: 0.402 ± 0.0006 cyc/deg vs. DM: 0.367 ± 0.002 cyc/deg; *P* < 0.0001) and CS (Ctrl: 8.66 ± 0.075 a.u. vs. DM: 5.84 ± 0.068 a.u., *P* < 0.0001) thresholds by seven weeks of hyperglycemia (Fig. 3A). In DM+Cont and DM+Wash mice, the decline in SF thresholds was halted at three weeks (*P* < 0.0001) and four weeks of treatment (*P* < 0.0001) whereas DM+Veh SF steadily worsened (Fig. 3A; Table). A similarly robust effect was seen in CS thresholds, with DM+Cont and DM+Wash mice having higher CS thresholds than DM+Veh after three (*P* < 0.0001) and four weeks (*P* < 0.0001) of L-DOPA treatment (Fig. 3B; Table). Although SF and CS were significantly protected from decline, DM+Cont mice had higher thresholds than DM+Wash for SF (*P* = 0.0248) and trending for CS (*P* = 0.0551) at the final timepoint, possibly indicating the start of diverging visual function protection from L-DOPA treatment.

### L-DOPA Treatment Maintained Retinal Vascular Function in the Diabetic Retina

**Figure 4.**
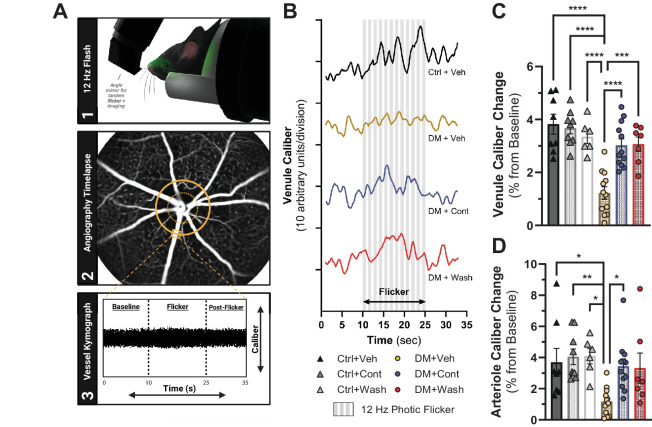
Flicker-induced retinal vasodilation improved with L-DOPA treatment in diabetic mice. **(A)** Graphical representation of in vivo neurovascular coupling imaging and analysis pipeline shows **(A1)** the set-up for stimulating the eye, **(A2)** the ICG-filled retinal blood vessels visualized with confocal scanning laser ophthalmoscopy, and **(A3)** a kymograph illustrating vessel dilation across time. **(B)** Representative individual venule caliber traces across pre-flicker, 15-second flicker (*gray bars*), and post-flicker period. **(C)** Averaged venule caliber percent change from baseline was significantly reduced in DM+Veh compared to control groups. Meanwhile, DM+Cont and DM+Wash mice showed significantly elevated venule response over DM+Veh. **(D)** Similarly, DM+Veh mice had significantly reduced arteriole caliber change compared to control groups. Although both DM+Cont and DM+Wash showed elevated arteriole caliber change over DM+Veh, only DM+Cont reached statistical significance. Ctrl+Veh (*n* = 8), Ctrl+Cont (*n* = 9), Ctrl+Wash (*n* = 6), DM+Veh (*n* = 12), DM+Cont (*n* = 12), and DM+Wash (*n* = 7). Data shown as mean ± SEM; *P < 0.05, **P < 0.01, **P < 0.01, ***P < 0.001, ****P < 0.0001. Includes assets from BioRender.

To assess the integrity of neurovascular coupling in the diabetic retina, the vasodilative response to increased neuronal demand elicited by 12 Hz photic stimulus was measured across retinal arterioles and venules using confocal scanning laser ophthalmoscopy (Figs. 4A, 4B). In response to light stimulus, Ctrl+Veh venules vasodilated 3.82% ± 0.38% whereas arterioles vasodilated 3.68% ± 0.89% from baseline vessel caliber (Figs. 4C, 4D). Arteriole vasodilation values matched flicker-induced observations in clinical^15^^,^^35^ and murine experiments.^36^^,^^37^ In addition, venule vasodilation mirrored recent reports of measurable venule dilative response by rodents downstream of functional hyperemia activity.^37^^–^^39^ As established in previous literature,^36^ DM+Veh light-evoked vasodilation decreased compared to Ctrl+Veh, by 68% in venules (*P* < 0.0001; Fig. 4C) and by 68.2% in arterioles (*P* = 0.0323; Fig. 4D) after 12 weeks of hyperglycemia.

After four weeks of continuous L-DOPA treatment, DM+Cont mice showed improvement over DM+Veh mice in both venule (*P* < 0.001) and arteriole (*P* = 0.0307) vasodilation (Figs. 4B–D; Table). Strikingly, after two weeks of L-DOPA treatment washout, DM+Wash mice also had post-treatment vascular protection as shown by greater venule vasodilation post-washout compared to DM+Veh (*P* = 0.0002; Figs. 4B, 4C; Table). Arteriole vasodilation showed similar trends for DM+Wash but did not reach statistical significance compared to DM+Veh (*P* = 0.124; Fig. 4D; Table).

### L-DOPA Induces Lasting Synapse-Related Transcriptional Change in Diabetic Mice

**Figure 5.**
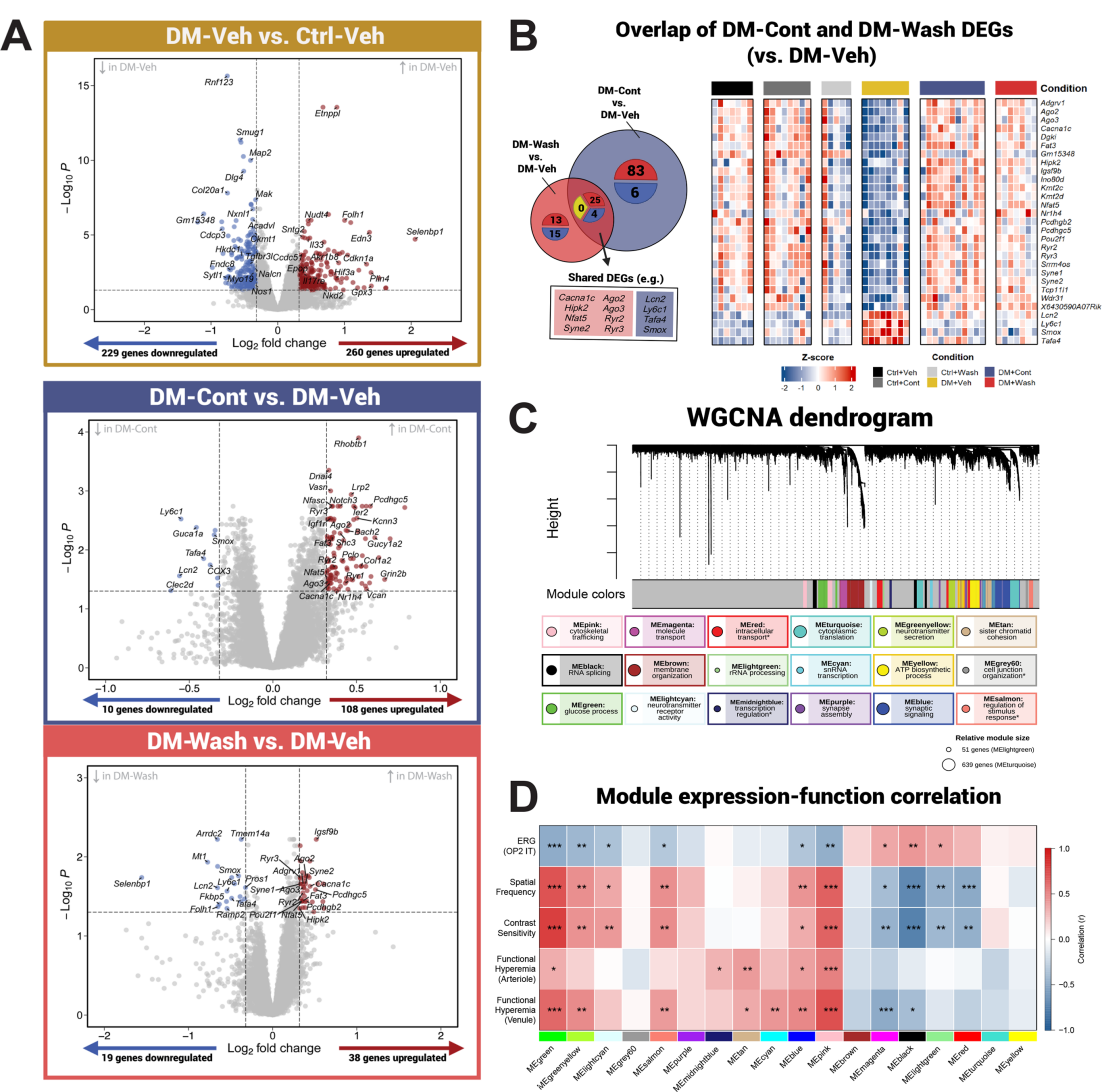
L-DOPA continued and washout treatment resulted in transcriptional changes that correlated with retinal function improvement. **(A)** DM+Veh mice had 260 upregulated and 229 downregulated DEGs compared to Ctrl+Veh mice; |log_2_ fold change| > 0.32, *P*.adj < 0.05). When comparing DM-Cont to DM-Veh groups, 108 upregulated and 10 downregulated DEGs were identified. When comparing DM-Wash to DM-Veh, 38 upregulated and 19 downregulated genes were highlighted (|log_2_ fold change| > 0.32, *P*.adj < 0.05). **(B)** Venn diagram of DM-Cont versus DM-Veh DEGs and DM-Wash versus DM-Veh DEGs, illustrates shared DEGs including upregulated genes (25) and downregulated genes (4) with no contra-regulated genes (0). Shared DEGs are represented in the heatmap, with columns representing individual samples grouped by condition and *z*-scores indicating individual gene expression relative to across-sample mean. **(C)** WGCNA highlighted 18 MEs across diabetic and L-DOPA treatment status. Below the dendrogram, each module is summarized in identity (overarching term from GO:BP) and size (relative circle size). *Asterisk* indicates modules without significant GO:BP terms. **(D)** WGCNA module-function correlation resulted in multiple relevant modules with significant correlations across all functional measures, including MEblue, MEpink, and MEgreen (|*r*| > 0.30, *P* < 0.05). Ctrl+Veh (*n* = 7), Ctrl+Cont (*n* = 8), Ctrl+Wash (*n* = 5), DM+Veh (*n* = 8), DM+Cont (*n* = 11), and DM+Wash (*n* = 7). Includes assets from BioRender.

To identify molecular changes driven by L-DOPA's post-treatment protective effects, transcriptional profiles across treatment groups were assessed in whole retinal tissue by bulk RNASeq and analyzed for correlation with functional measures. PCA identified nearly 45% of variance across first and second principle components, with PC2 representing significant separation of DM+Veh from other treatment groups (Supplementary Fig. S3). Among 13,665 genes retained after filtering, 489 DEGs (229 downregulated, 260 upregulated: Fig. 5A) were identified in DM-Veh whole retina compared to Ctrl-Veh, outlining transcriptional changes due to the DM model. With L-DOPA treatment, 118 DEGs (10 downregulated, 108 upregulated) were identified in DM-Cont retina compared to DM-Veh, whereas only 57 DEGs (38 upregulated, 19 downregulated) were identified in DM-Wash compared to DM-Veh (Fig. 5A). Over 50% of DM+Wash DEGs identified were shared with DM-Cont DEGs and maintained the same direction of regulation (Fig. 5B). Although L-DOPA-treated DM mice still shared partial gene expression similarity with DM+Veh, these cross-treatment DEGs showed similar expression to controls, reflecting L-DOPA partially modulates gene expression as opposed to supporting complete disease reversal (Supplementary Fig. S4, Supplementary Table S1, Supplementary Data S1–S2). This is supported by L-DOPA-treated DM mice compared to DM+Veh, showing minimal differential gene expression representation of inflammatory and oxidative stress pathways known to be relevant to DR pathophysiology (Supplementary Fig. S5, Supplementary Data S3).

WGCNA was used to identify modules of co-expressed genes across treatment groups that were correlated with retinal functional outcomes. WGCNA identified 18 modules, or clusters of genes co-expressed across retinal samples (Fig. 5C). These modules were then described by top GO terms related to biological processes (e.g., glucose process, synapse assembly, transcription regulation).

**Figure 6.**
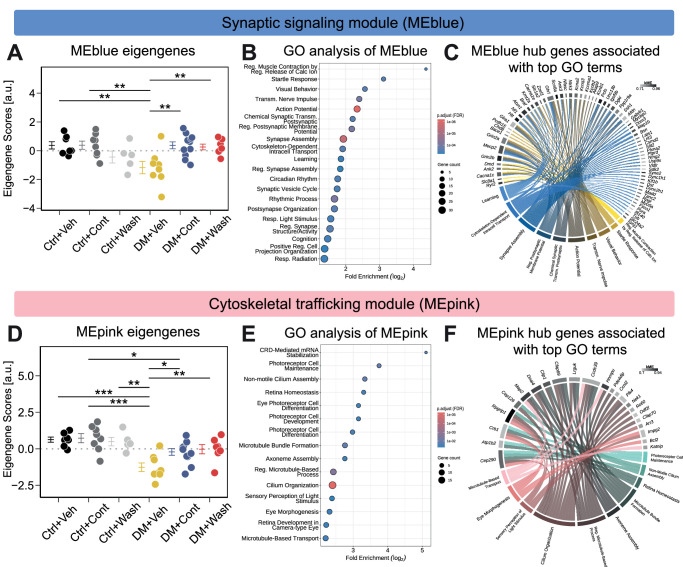
WGCNA modules sensitive to L-DOPA treatment in diabetic mice reflect changes in neuronal signaling, synapses, and cell maintenance. **(A)** Within MEblue, the ME significantly differed between DM+Veh versus DM+Cont and DM+Wash groups (mean ± SEM, linear model, *P*.adj < 0.05). **(B)** GO analysis for biological processes of relevant MEblue genes (kME > 0.60) identified signaling and plasticity-related GO terms (FDR-adjusted *P* < 0.05). **(C)** Chord diagram representing MEblue hub genes (kME > 0.70) shows associations to top 10 enriched GO terms (>2 gene count). Chord color indicates unique GO terms and connection with module-specific genes, with chord color labeled per GO term in the outer ring. Associated gene kME is shown as a separate grayscale bar and labeled per gene in the outer ring. **(D)** Similar to MEblue, MEpink significantly differed in DM-Cont and DM-Wash compared to DM-Veh groups (mean ± SEM, linear model, *P*.adj < 0.05). **(E)** GO analysis for biological processes of relevant MEpink genes (kME > 0.60) identified GO terms related to cytoskeletal dynamics and ciliary function (*P*.adjust < 0.05). **(F)** Chord diagram representing MEpink hub gene (kME > 0.70) illustrates associations with top 10 enriched GO terms (>2 gene count). Ctrl+Veh (*n* = 7), Ctrl+Cont (*n* = 8), Ctrl+Wash (*n* = 5), DM+Veh (*n* = 8), DM+Cont (*n* = 11), and DM+Wash (*n* = 7). Includes assets from BioRender.

Within each module, the ME (first principal component of principal component analysis) represents gene expression of co-varying genes per module. When individually correlated with the final week of retinal functional assessments across ERG, OMR, and FH, modules MEgreen, MEblue, and MEpink maintained significant correlation across all functional measures (Fig. 5D). Within these modules, only MEblue and MEpink modules were significantly different between DM+Veh and DM+Cont/Wash groups and thus treatment-sensitive (Figs. 6A, 6D). Notably, no modules were significantly different between DM+Cont and DM+Wash. Visualizing gene expression specific to these modules supported ME patterns, showing predominant gene downregulation in DM+Veh and control-like expression patterns in DM+Cont and DM+Wash (Supplementary Fig. S6, Supplementary Data S1). Furthermore, DEGs from initial L-DOPA-treated DM vs. DM+Veh comparisons reflected enrichment of general GO terms pertaining to treatment-sensitive modules (Supplementary Table S2, Supplementary Data S7).

GO analysis of MEblue-specific hubgenes (>0.7 kME; Supplementary Data S4) revealed top terms involving synapse signaling and neurotransmission (e.g., action potential, synapse structure and activity), as well as sensory and rhythmic processes (e.g., response to light stimulus, circadian rhythm) (Figs. 6A–C). Interestingly, 20 MEblue hubgenes (>0.7 kME) (e.g., calcium voltage-gated channel subunit alpha 1 a, *Cacna1c;* ryanodine receptor 2*, Ryr2*) identified as overlapping DEGs shared between DM-Cont versus Veh and DM-Wash versus DM-Veh comparisons (Fig. 6C). Given the dysregulation of calcium influx in diabetic retinopathy,^40^^–^^42^ this shift toward homeostatic expression of calcium channel genes in DM+Cont/Wash suggests L-DOPA-mediated transcriptional stabilization related to calcium signaling. Cell-type percent enrichment analysis of MEblue hubgenes reflected limited (<10%) cell-specific identity, with modest enrichment distributed across neuronal (e.g., bipolar, horizontal, rods, cones) and vascular components (e.g., pericytes and endothelial cells) (Supplementary Fig. S7, Supplementary Data S5, S6). Alongside correlation with improvement in diabetic retinal function, this module consists of a broad and maintained change of synaptic and calcium-associated gene expression in the diabetic retina after L-DOPA treatment.

**Figure 7.**
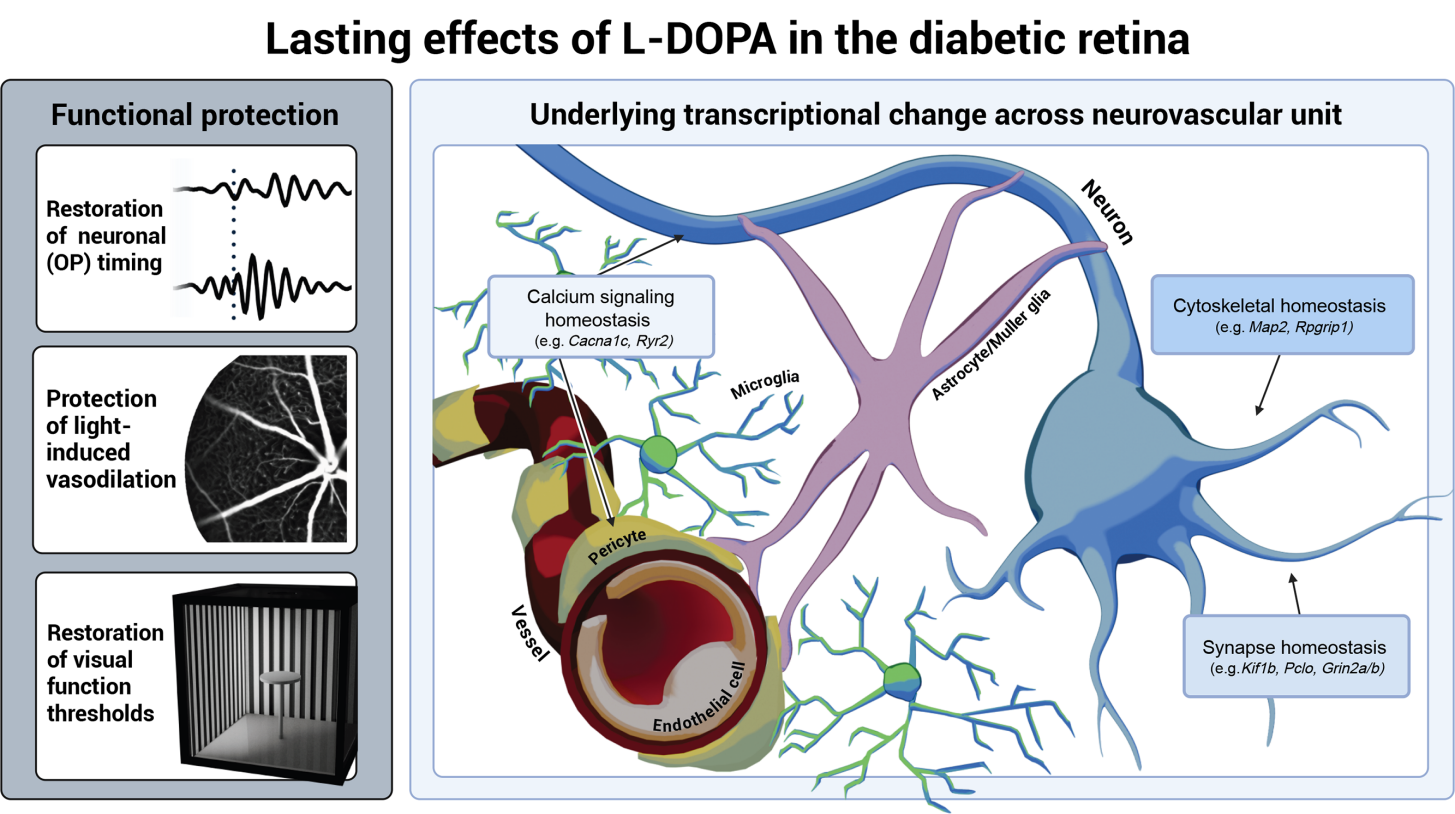
Visual summary of the lasting protective effects of L-DOPA on the diabetic retina across changes in function and associated gene transcription.

Furthermore, the MEpink module suggested L-DOPA-associated regulation of cytoskeletal components in the diabetic retina. GO analysis of MEpink hubgenes (>0.7 kME; Supplementary Data S4) highlighted terms relevant to (1) retinal maintenance (e.g., retina homeostasis, photoreceptor cell maintenance), (2) cilium and axoneme organization (e.g., cilium organization, axoneme assembly), and (3) microtubule-based processes (e.g., microtubule-based transport, microtubule bundle formation) (Figs. 6D–F). Notable MEpink hubgenes associated with top GO terms included critical regulators in microtubule stability and dynamics *(*microtubule-associated protein 2*, Map2;* CAP-Gly domain containing linker protein 1, *Clip1)*,^43^^–^^45^ as well as cAMP-signaling and anti-apoptotic survival factors (phosphodiesterase 4D interacting protein, *Pde4dip;* B-cell lymphoma 2, *Bcl2)*^46^^,^^47^ (Fig. 6F). Similar to the previous module, DEGs resolved from initial L-DOPA-treated DM versus DM+Veh comparisons. Although cell-type percent enrichment analysis of MEpink hubgenes was similarly lacking in a cell-specific identity (<12%), enrichment was primarily neuronal and photoreceptor-driven across rods and cones (Fig. 7, Supplementary Data S5, S6). Together, this module supports early cytoskeletal remodeling observed in the diabetic retina^48^^,^^49^ and suggests a shift to control-like homeostatic expression of cytoskeletal maintenance genes after L-DOPA treatment.

## Discussion

This study demonstrated L-DOPA-mediated mitigation of early functional deficits across the NVU in the diabetic retina and investigated the underlying transcriptional alterations associated with its post-treatment protective effects. Gene network analysis correlated with L-DOPA-induced improvement of diabetic retinal function, suggesting L-DOPA affects gene networks surrounding synaptic regulation and cytoskeletal maintenance (Fig. 7).

### L-DOPA's Post-Treatment Neurovascular Protection in the Diabetic Retina

L-DOPA's short half-life is a core limitation to its use as a therapeutic strategy in disorders like Parkinson's disease.^30^ Even when paired with decarboxylase inhibitors to minimize peripheral metabolism, L-DOPA is found in plasma and cerebrospinal fluid for at most a few hours.^50^ We hypothesize that for L-DOPA to have functional protection beyond its expected pharmacokinetic window, its effect stretches across multiple aspects of the interdependent NVU and may even be independent of dopamine metabolism.

Novel to this study, L-DOPA treatment showed restoration of light-induced retinal vasodilation, supporting the neuroprotective effects of L-DOPA across NVU dysfunction in early DR. While dopamine is typically described as a retinal neuromodulator, it also acts on vascular and glial components of the NVU. Dopamine receptors from both D1-like (D1R, D5R) and D2-like (D2R, D3R, D4R) families are broadly expressed across most major retinal cell types, including endothelial cells and pericytes. In pericytes, dopamine activation of ATP-sensitive potassium currents serves as a mechanism of metabolic sensing and regulation of vascular tone.^51^ Dopamine has also been associated with anti-angiogenic properties in the retina.^52^^–^^54^ Together, these observations support plausible means of L-DOPA-derived modulation of retinal vascular responses.

Alternatively, reciprocal signaling across the NVU is well-established in retinal pathology.^55^^–^^57^ For instance, neuronal *Nrf2* activation in the ischemic retina promotes revascularization through paracrine crosstalk with endothelial cells.^58^ Moreover, inhibition of pericyte function through PDGFRβ blockage has been shown to impair Muller glia response to light-induced retinal injury.^55^ Coordinated improvements across neurovascular function with L-DOPA treatment could reflect similar NVU-based reciprocal signaling, suggesting a protective feedback loop that spans retinal cell types and supports protection beyond transient neurotransmitter-receptor interactions.

Separately, recent research on L-DOPA's role in the melanin synthesis pathway has established 1) L-DOPA is an endogenous ligand to GPR143, a G-protein coupled receptor found in the retinal pigment epithelium (RPE),^59^^,^^60^ and 2) L-DOPA benefits retinal and visual function in a rodent albinism model lacking functional tyrosinase for synthesizing L-DOPA in the RPE.^61^ Although L-DOPA treatment restores reduced retinal dopamine levels in diabetic rodent models,^8^^,^^9^ this research highlights the possibility that the lasting effect of L-DOPA treatment in the diabetic retina could be supported downstream of L-DOPA itself and not exclusively by increased dopamine levels.

### Gene Network Changes Correlate With L-DOPA-Induced Neuroprotection in DR

Although L-DOPA's functional protection spans diabetic rodent models and clinical patients, the biology underlying its protection has been unclear. Using WGCNA, modules with functional correlation showed heterogenous, cross-NVU identity with limited retinal cell-type enrichment, and notably, none were sensitive to L-DOPA treatment duration. This suggests preservation of protective gene network changes during washout rather than engaging a separate, novel transcriptional program.

Within the synaptic signaling module (MEblue), L-DOPA treatment was associated with concerted homeostatic expression of signaling and synapse-related genes that correlated with improved retinal function. Prominent among these were genes relevant to intracellular calcium regulation across neurons, glia, and vasculature, including voltage-gated calcium channels and ryanodine receptors (e.g., *Cacna1c, Ryr2*). Dysregulation of calcium signaling is a part of DR's early pathology, including reduced calcium signaling in presynaptic GABAergic amacrine cells,^40^ as well as hyperglycemia-induced excitotoxic calcium overload,^62^^,^^63^ upregulation of low-voltage T-type calcium channels,^64^ and delayed calcium buffering.^62^ In this context, our findings suggest a nuanced role of L-DOPA on calcium channels in the diabetic retina, potentially restoring balanced calcium influx and cycling to support synaptic function and homeostasis.

Beyond calcium signaling, the synaptic signaling module spanned other integral genes supporting synapse structure and performance. These included motor proteins that support synaptic function across retinal neurons (e.g., kinesin family member 1b, *Kif1b;* myosin VA*, Myo5a*),^65^^,^^66^ glutamatergic receptor components crucial to synaptic plasticity and enriched in amacrine and retinal ganglion cells (e.g., glutamate ionotropic receptor NMDA type subunit 2A and 2B, *Grin2a* and *Grin2b*),^67^ and scaffolding proteins essential for ribbon synapse organization and neurotransmitter release at photoreceptor and bipolar cell synapses (e.g., piccolo presynaptic cytomatrix protein, *Pclo;* bassoon presynaptic cytomatrix protein*, Bsn*).^68^^,^^69^ Given prior observations of synapse-related gene downregulation in diabetic rodents,^67^^,^^70^^–^^72^ coordinated regulation of these synapse-relevant genes suggests that L-DOPA enhances synaptic resilience, with downstream benefit for NVU integrity by stabilizing neuronal activity and reducing stress on glial and vascular components.

L-DOPA sensitivity and correlation with NVU functional improvement was also observed in a cytoskeletal trafficking module (MEpink). Core to this module were microtubule-associated genes (e.g., *Map2, Clip1, Pde4dip*), reflecting L-DOPA-induced coordinated regulation of microtubule dynamics, intracellular trafficking, and cellular migration that supports dendritic and synaptic architecture.^43^^–^^45^^,^^47^ Notably, *Map2* is known to be reduced in the diabetic retina, indicating early retinal neurodegeneration linked to cytoskeletal dysfunction.^48^^,^^73^ The same module was also enriched for ciliary genes (e.g., RPGR interacting protein 1, *Rpgrip1;* centrosomal protein 290*, Cep290;* Bardet-Biedl syndrome 1*, Bbs1*) essential to photoreceptor maintenance and function,^74^^–^^76^ contributing to the module's photoreceptor-specific neuronal enrichment. Collectively, coordinated regulation of microtubule and ciliary gene expression alongside calcium and synaptic signaling genes support a model in which L-DOPA promotes structural and functional plasticity under hyperglycemic stress, strengthening communication across the NVU and contributing to protection of retinal function.

## Conclusions

This work established post-treatment neurovascular protection by L-DOPA in the diabetic mouse retina, with correlated gene expression highlighting a potential role of L-DOPA in supporting homeostatic synaptic signaling and cytoskeletal maintenance. Given L-DOPA's existence as a repurposed drug that provides post-treatment benefit to neurovascular components vulnerable to hyperglycemia in the retina, it maintains potential as a clinical treatment option to prevent vision-threatening DR. Limitations of this study include its specificity for early stages of DR without proliferation, as well as close proximity of washout to L-DOPA treatment window that may be confounded by lasting symptomatic thresholds previously observed in Parkinson's disease (PD).^77^ Further, while non-PD studies across DR^10^^,^^78^ and age-related macular degeneration^79^ suggest short-term tolerability of L-DOPA, larger and longer clinical studies are needed to define safety, dosing, and efficacy with chronic L-DOPA treatment. Future research on L-DOPA's post-treatment effect in the diabetic retina should resolve its temporal limits, assessing L-DOPA's relevance in modulating disease trajectory and efficacy at later DR stages. Other future directions include confirming protein-level changes with cell specificity, assessing potential sex-specific L-DOPA pharmacokinetics,^80^ and determining L-DOPA's primary metabolic pathway.

## Supplementary Material

Supplement 1

Supplement 2

Supplement 3

Supplement 4

Supplement 5

Supplement 6

Supplement 7

Supplement 8
