# Supplementary material for: L-DOPA Promotes Post-Treatment Neurovascular and Synaptic Homeostasis in Early Diabetic Retinopathy
Source: Invest Ophthalmol Vis Sci. 2026 Jul 20;67(8):42. doi: 10.1167/iovs.67.8.42 (PMC13398881; doi:10.1167/iovs.67.8.42)
Supplement: Supplement 1 [file iovs-67-8-42_s001.docx]

**Supplementary materials**

**
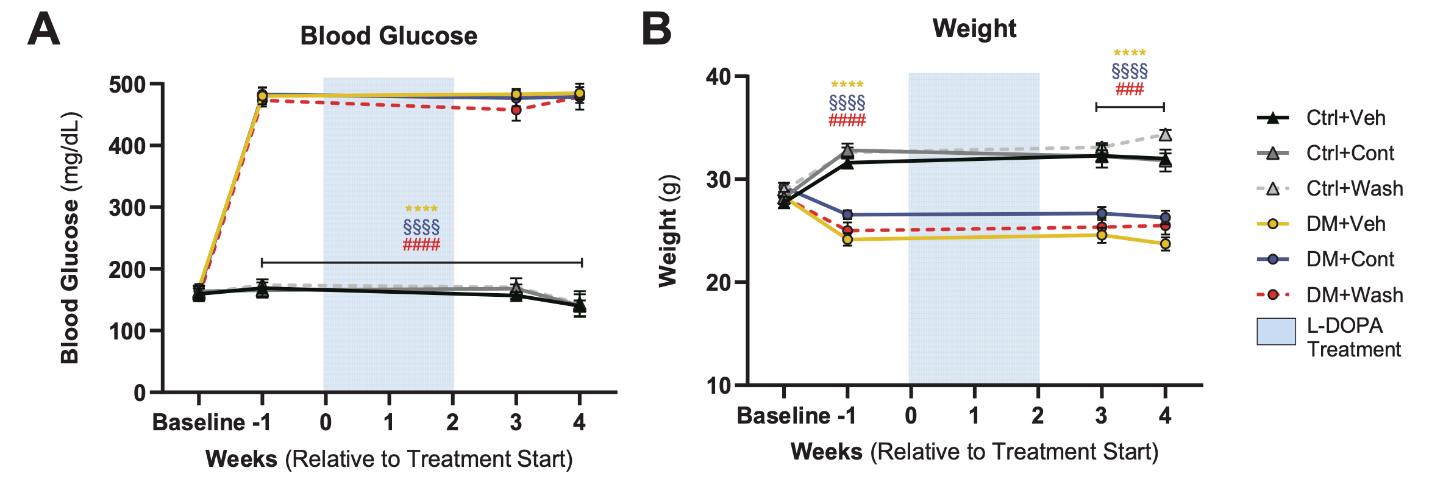
**

**Fig. S1.** Diabetic mice showed consistent blood glucose elevation and lack of weight gain. (**A**) Blood glucose (mg/dL) was significantly elevated in diabetic mice prior to L-DOPA treatment (-1 wk treatment), as well as after treatment (3-4 week treatment). Control mice did not show BG elevation with vehicle or treatment. (**B**) All diabetic mice maintained significantly lower body weight than control counterparts, with weight consistent within groups across treatment time period. Data shown as mean ± SEM; (*) DM+Veh vs. Ctrl+Veh, (§) Ctrl+Veh vs. DM+Cont, (#) Ctrl+Veh vs. DM+Wash; ****p* < 0.001,*****p* < 0.0001.

**
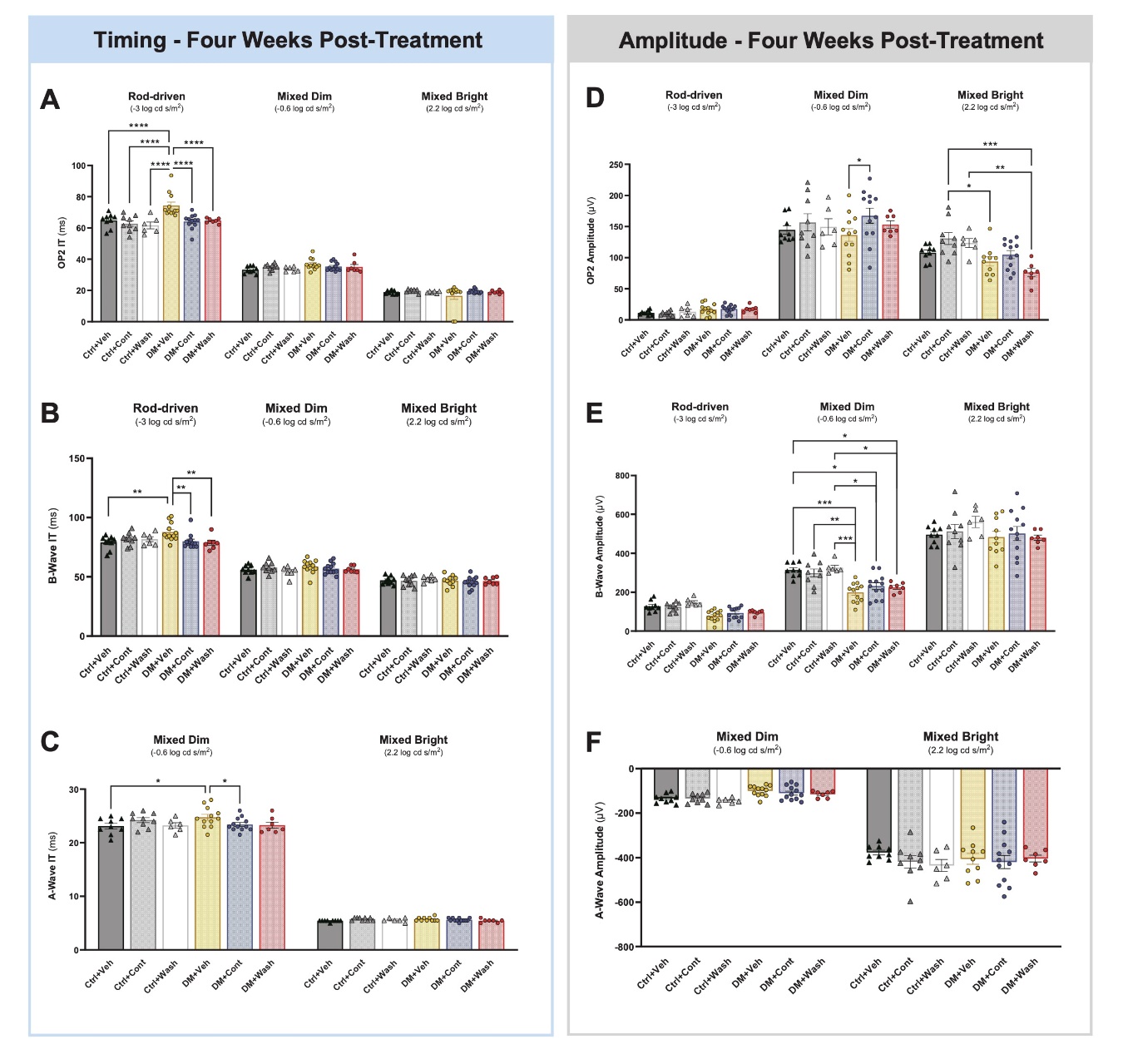
**

**Fig. S2.** Rod-driven oscillatory potential timing was the primary L-DOPA-sensitive ERG component in diabetic mice. (**A**) Rod-driven oscillatory potential implicit timing was elevated in DM+Veh only. (**B**) Rod-driven b-wave implicit timing showed a similar specificity for delay in DM+Veh mice, but without significant differences compared to Ctrl+Cont and Ctrl+Wash. (**C**) Limited to mixed (rod/cone) dim and mixed bright conditions, mixed dim a-wave timing appeared delayed in DM+Veh mice compared to Ctrl+Veh and DM+Cont mice. (**D**) Oscillatory potential amplitude differences appeared only under mixed dim and mixed bright conditions, without consistent differences between DM+Veh and control mice. (**E**) Mixed dim b-wave amplitude was reduced in DM+Veh mice compared to controls, as well as DM+Cont and DM+Wash mice compared to Ctrl+Veh and Ctrl+Wash. (**F**) No significant differences in A-wave amplitude under mixed dim and bright conditions were seen across diabetic and treatment groups. Data shown as mean ± SEM; *p<0.05, **p<0.01,****p* < 0.001,*****p* < 0.0001.


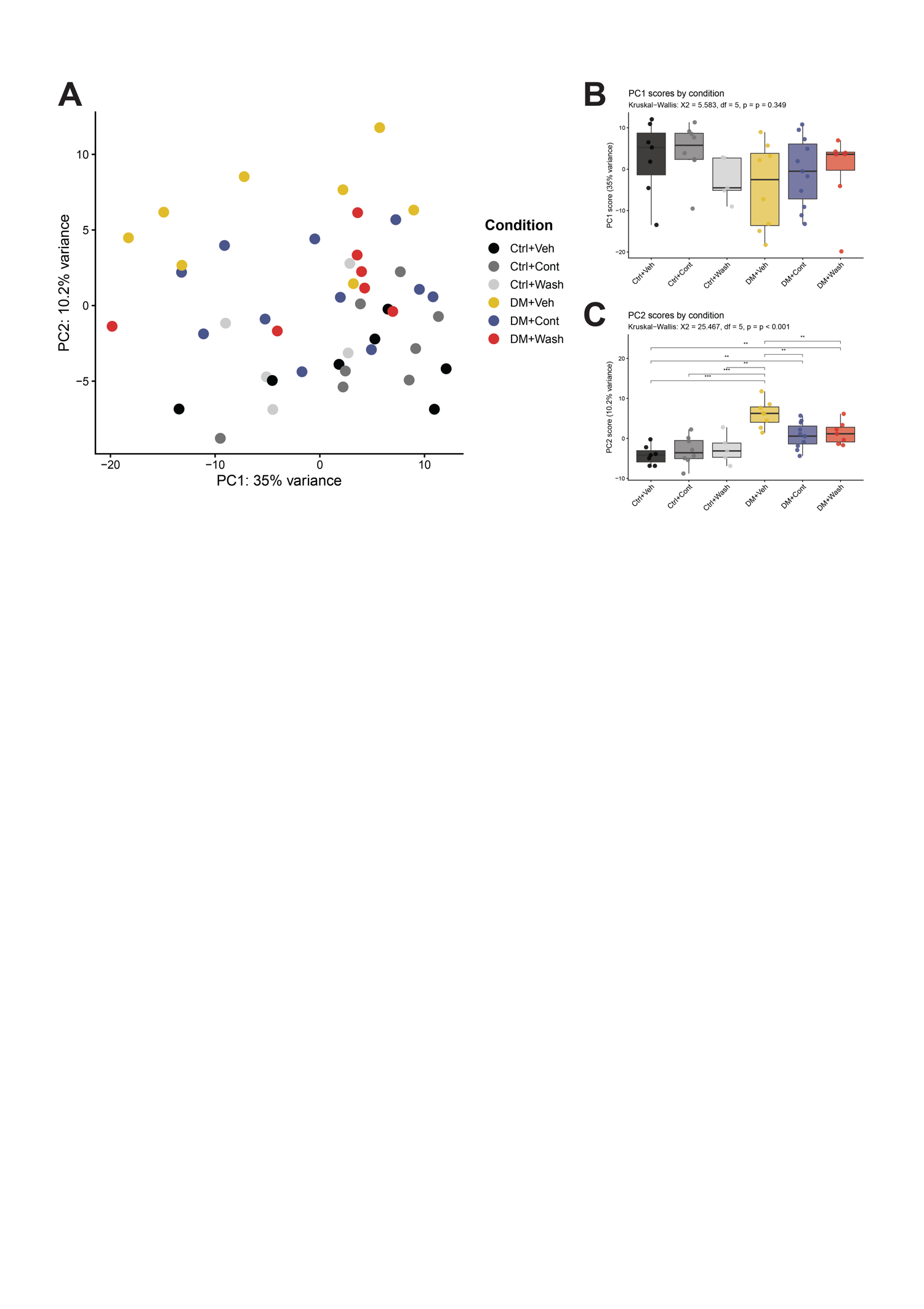


**Fig. S3.** PCA of bulk transcriptomic profiles across experimental groups. (**A**) Principle component analysis of RNAseq count data, with PC1 accounting for 35.0% total variance and PC2 accounting for 10.2% variance. (**B, C**) PC1 and PC2 scores summarized with boxplots of individual samples. Global differences within PC1 and PC2 scores were assessed with Kruskal-Wallis tests, with significant pairwise comparisons evaluated using Wilcoxon rank-sum tests with Benjamini-Hochberg correction. PC1 showed no significant global condition effect, while PC2 showed a significant condition-associated separation. Boxplots shown as median; **p<0.01,****p* < 0.001.


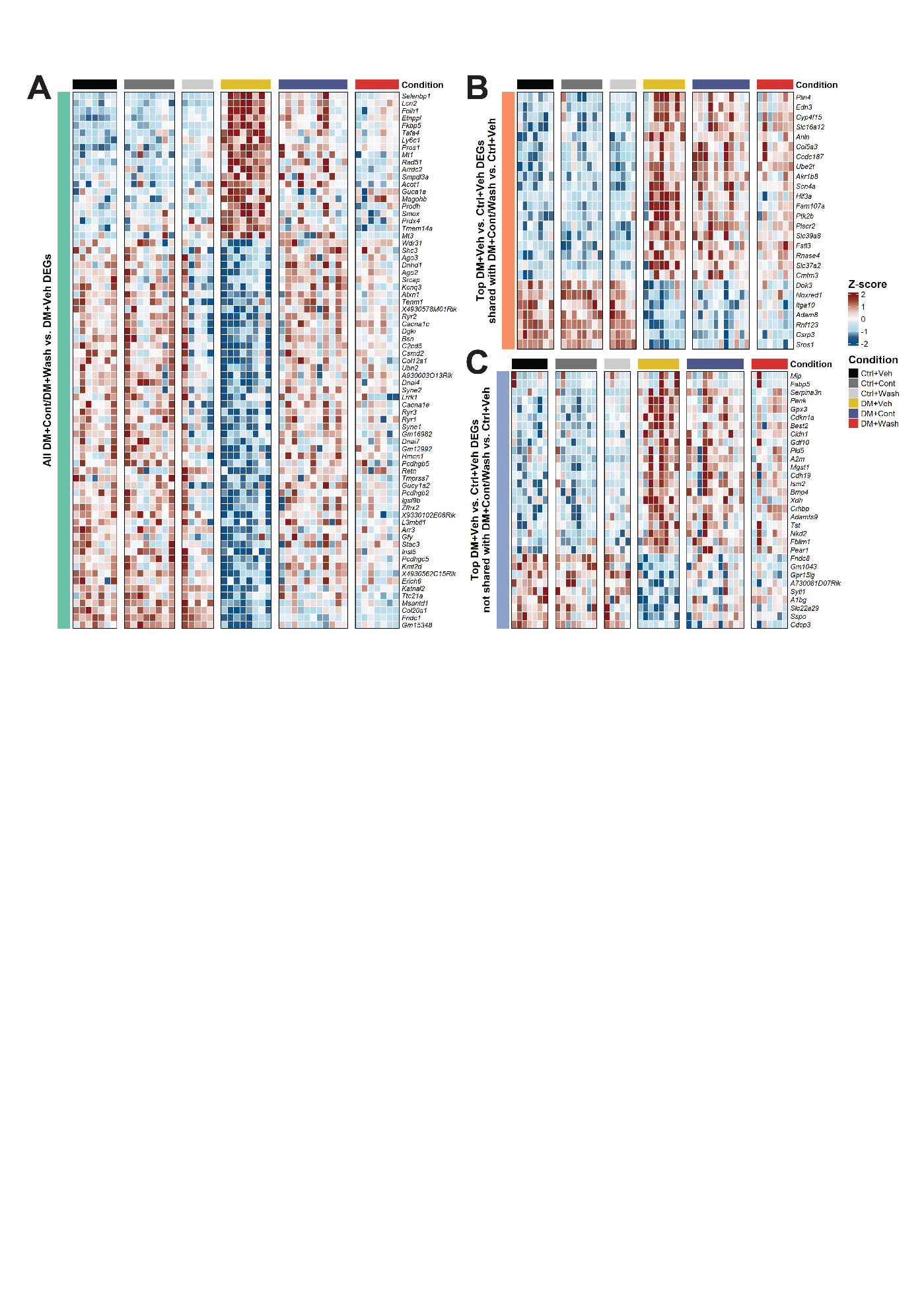


**Fig. S4.** Gene expression patterns among selected DEG subsets. (**A**) All genes differentially expressed in DM+Cont or DM+Wash relative to DM+Veh; (**B**) top (|log2FC|) DM+Veh vs. Ctrl+Veh DEGs shared with DM+Cont/DM+Wash vs. Ctrl+Veh DEGs; (**C**) top (|log2FC|) DM+Veh vs. Ctrl+Veh DEGs not shared with DM+Cont/DM+Wash vs. Ctrl+Veh DEGs. Columns represent individual samples grouped by condition. Z-scores indicate individual gene expression’s relationship to associated across-sample mean. All DEGs meet filtering criteria of Benjamini-Hochberg adjusted p-value (<0.05) and |log2FC| (>0.32).


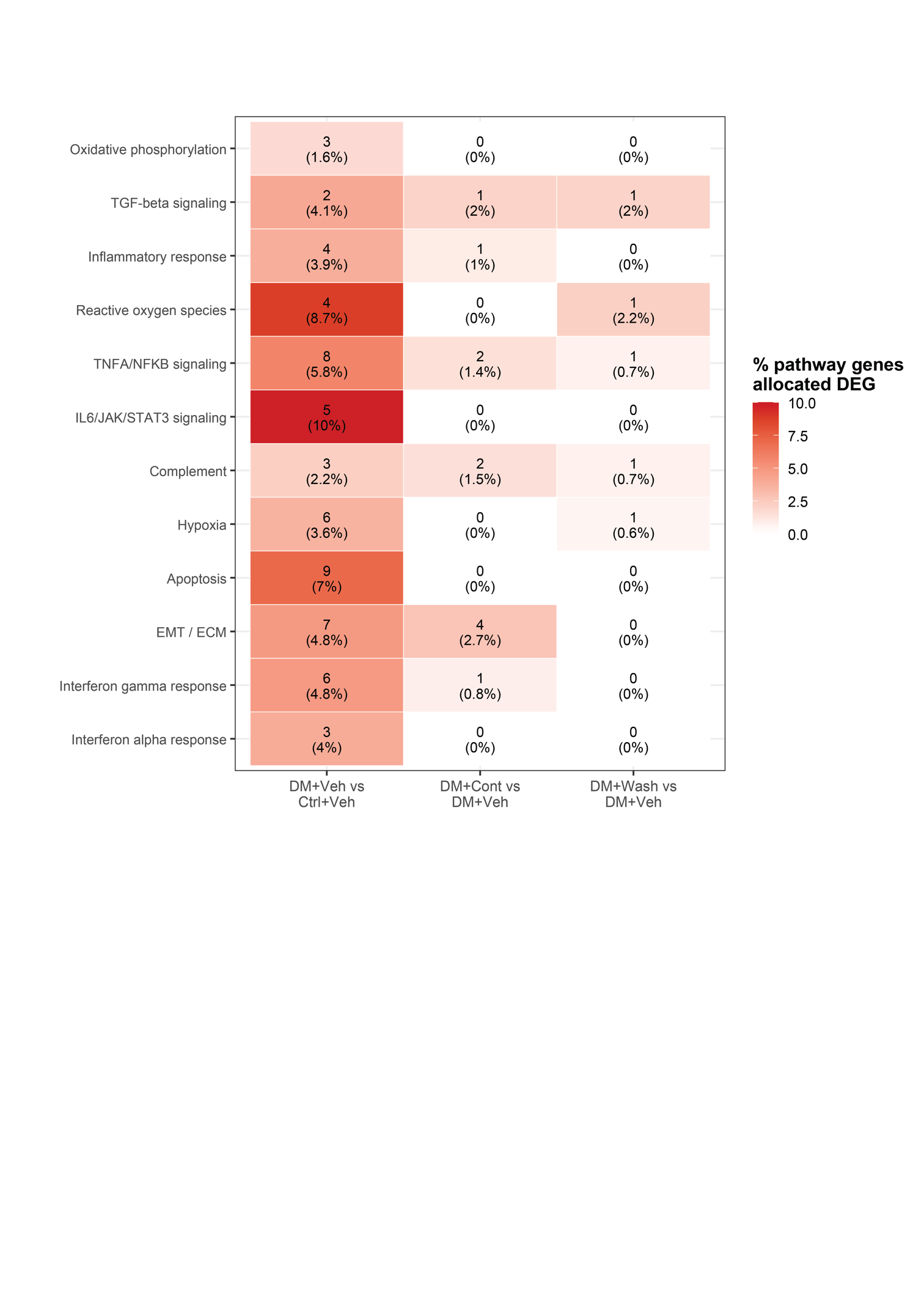


**Fig. S5.** Overlap of hallmark oxidative stress and inflammatory genes with DEGs. Selected MSigDB Hallmark pathways reflecting inflammation and oxidative stress genes were assessed for enrichment among DEGs from DM+Veh vs. Ctrl+Veh, DM+Cont vs. DM+Veh, and DM+Wash vs. DM+Veh comparisons. Tile numbers indicate count of DEGs overlapping with pathway, with percentage of hallmark genes represented in comparison-specific DEGs shown in parentheses and by tile color. Results highlight broader inflammation and oxidative stress pathway representation in the DM+Veh vs. Ctrl+Veh comparison, with minimal DEG overlap with DM+Cont and DM+Wash treatment comparisons.


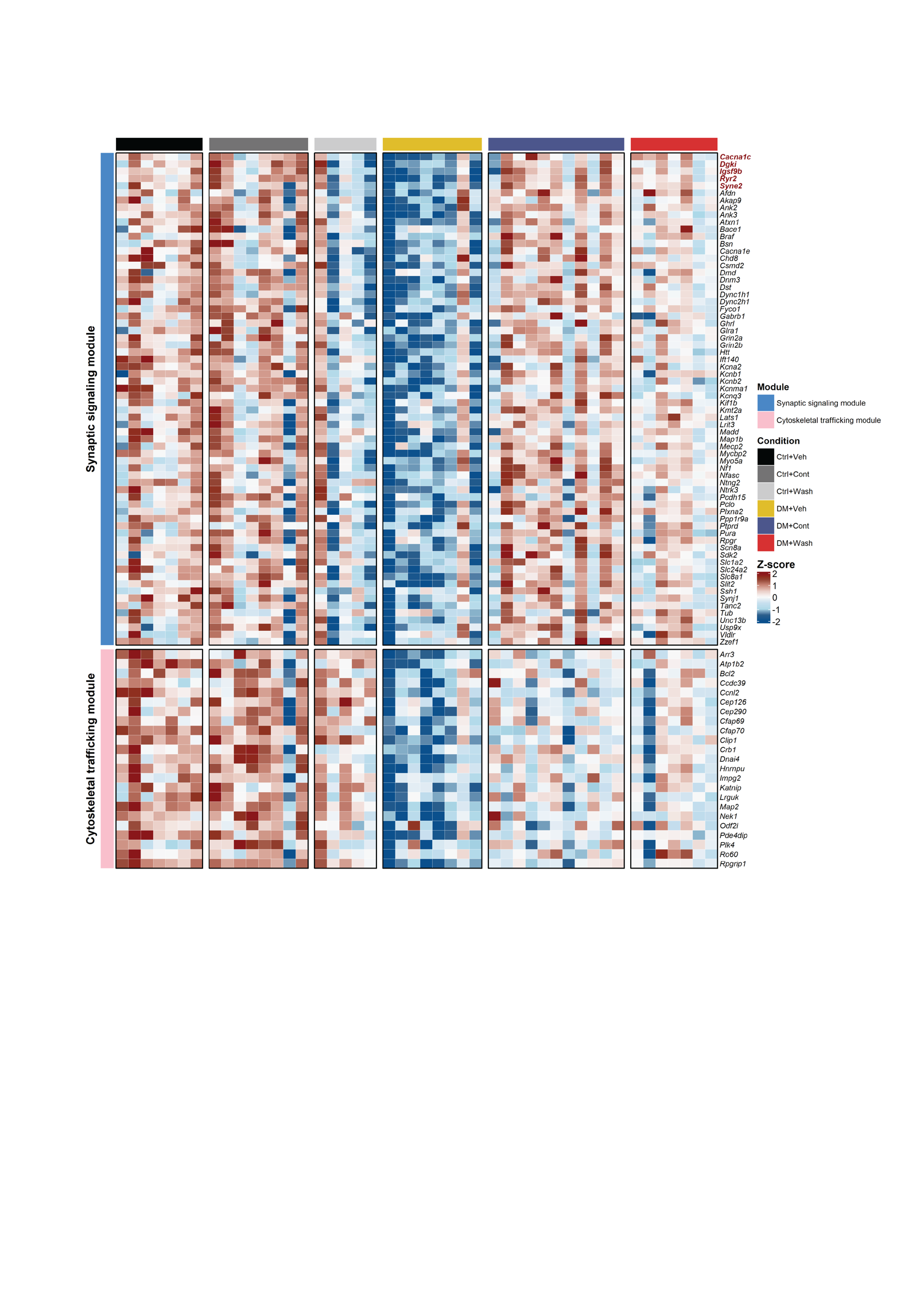
 **Fig. S6.** WGCNA hubgene expression across diabetic and treatment conditions. Gene expression heatmap of select WGCNA-identified hubgenes, representing genes from synaptic signaling and cytoskeletal trafficking modules that had high module identity (>0.7 kME) and overlapped with module-specific gene ontology terms containing at least three module genes. Columns represent individual samples grouped by condition, and rows represent module-associated hubgenes. Bold red gene labels denote hubgenes overlapping with DEGs shared by DM+Cont vs. DM+Veh and DM+Wash vs. DM+Veh comparisons.


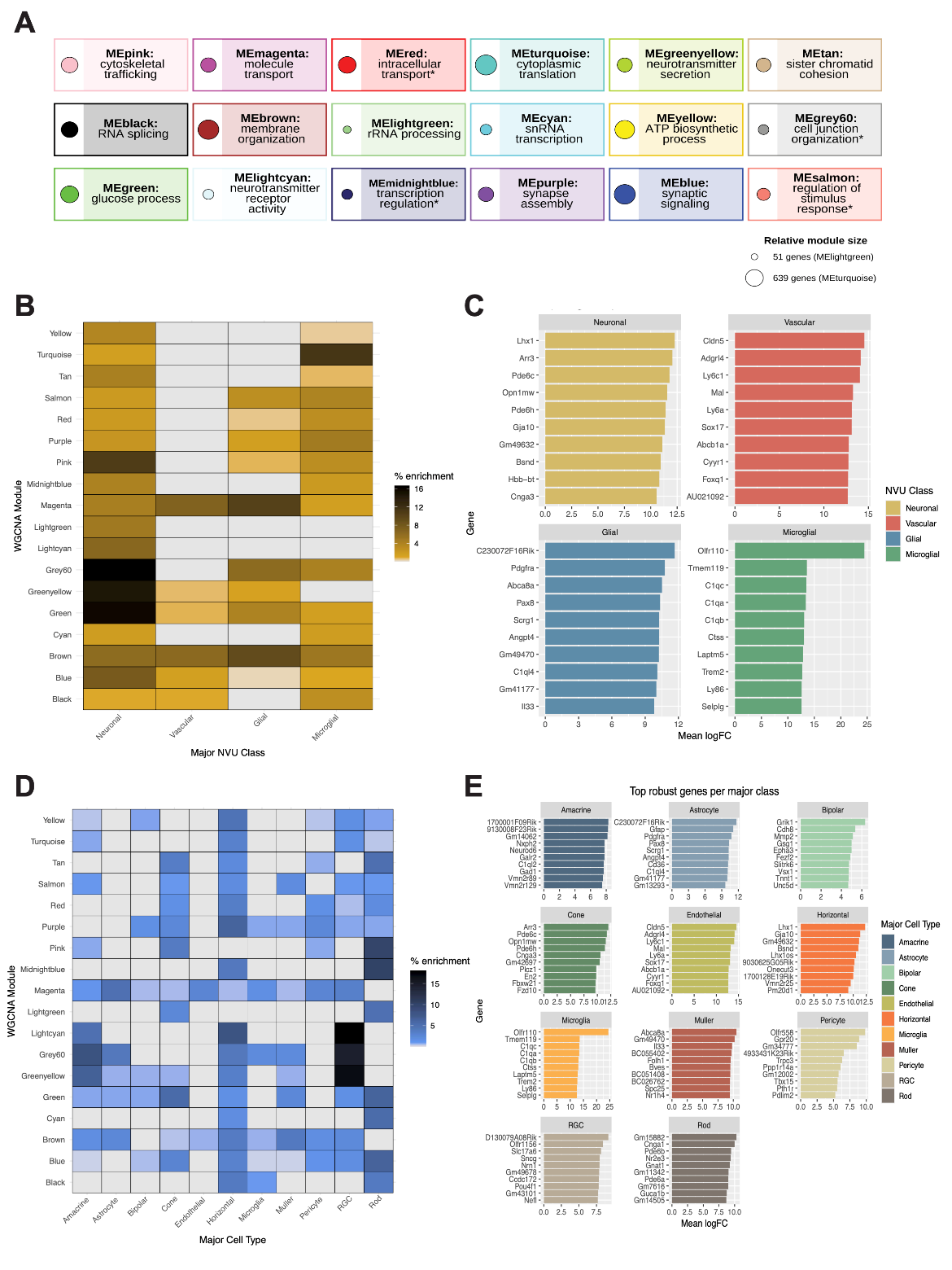


**Fig. S7.** **Cell-type enrichment and neurovascular unit class enrichment was limited across WGCNA modules.** (**A**) WGCNA modules with summary identity (overarching term from GO:BP) and size (relative circle size). Asterisk indicates modules without significant GO:BP terms. (**B**) Heatmap of major NVU class percent enrichment across WGCNA modules, with a neuronal enrichment across all modules alongside heterogenous spread of vascular, glial, and microglial enrichment. (**C**) Top 10 genes per NVU class, filtered from top 2000 genes unique to each NVU class (logFC > 0.25, padj < 0.05). (**D**) Heatmap of major retinal cell type percent enrichment within WGCNA modules. (**E**) Top 10 genes per major cell class, filtered from top 1000 genes unique to each major retinal cell class.


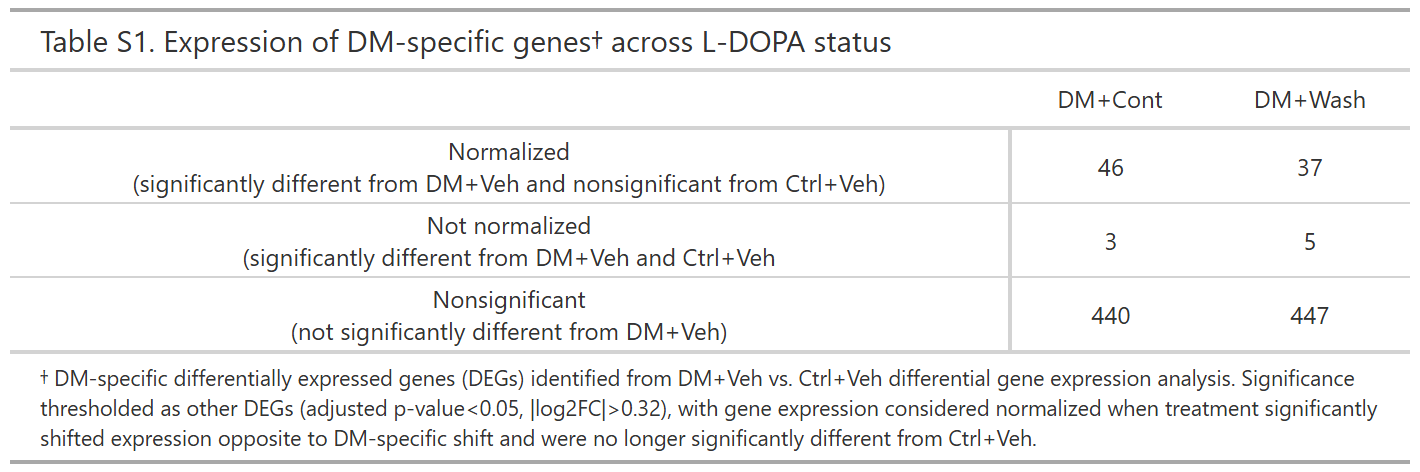


**Table S1.** Expression of DM-specific genes across L-DOPA status. DM-specific DEGs identified from DM+Veh vs. Ctrl+Veh comparison were categorized according to their expression status in DM+Cont and DM+Wash groups. Genes were considered significantly reversed and control-like when treatment significantly shifted expression opposite to the DM-associated direction and the treatment group was no longer significantly different from Ctrl+Veh. Genes were considered significantly different but not control-like when treatment significantly altered expression and were significantly different from Ctrl+Veh expression. Remaining genes were classified as nonsignificant. All DEGs identified with adjusted p < 0.05 and |log2FC| > 0.32.


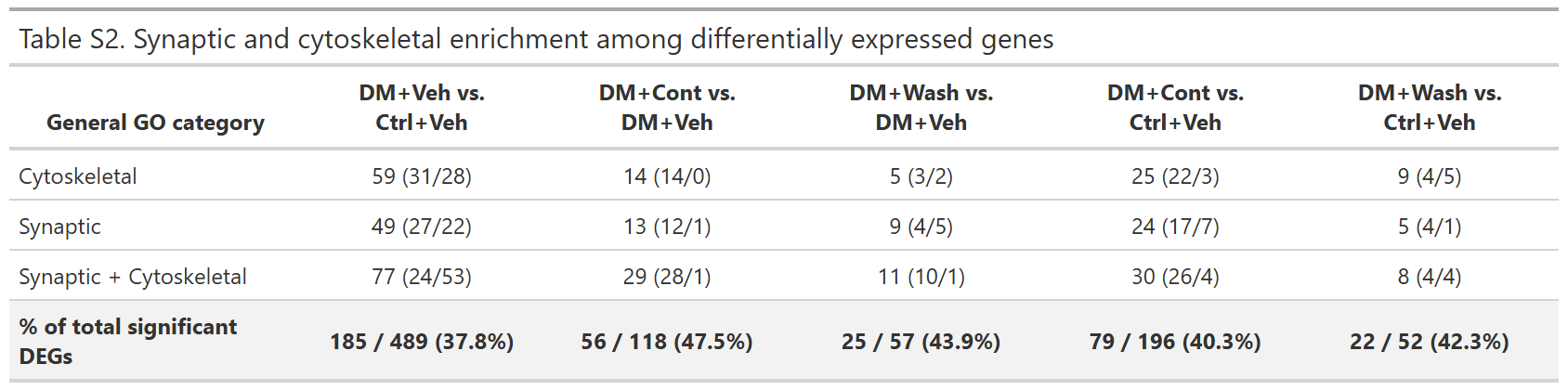


**Table S2.** Synaptic and cytoskeletal enrichment among differentially expressed genes. Significant DEGs identified from pairwise comparisons were annotated with Gene Ontology Biological Processes (GO:BP) terms related to synaptic structure/function and cytoskeletal organization/function and collapsed into general Synaptic, Cytoskeletal, or Synaptic + Cytoskeletal groups. Values represent the total number of unique DEGs in each comparison, with the number of upregulated and downregulated genes shown in parentheses (up/down). Percentages reflect total significant DEGs represented by highlighted GO:BP categories out of total DEGs per comparison.

**Data S1.** (separate file) Differentially expressed genes list compiled across DM+Veh, DM+Cont, DM+Wash, and Ctrl+Veh comparisons.

**Data S2.** (separate file) Differentially expressed genes from DM+Veh vs. Ctrl+Veh with control-like status across treatment.

**Data S3.** (separate file) Overlap of hallmark inflammation and oxidative stress pathways with differentially expressed genes.

**Data S4.** (separate file) WGCNA synaptic signaling and cytoskeletal trafficking module genes and associated gene ontology.

**Data S5.** (separate file) Retinal gene list for identification of neurovascular unit enrichment.

**Data S6.** (separate file) Retinal gene list for identification of major cell class enrichment.

**Data S7.** (separate file) Annotation of synaptic and cytoskeletal biological processes across differentially expressed genes.
